# Is Visual Registration Equivalent to Semiautomated Registration in Prostate Biopsy?

**DOI:** 10.1155/2015/394742

**Published:** 2015-03-02

**Authors:** Jin Tae Kwak, Cheng William Hong, Peter A. Pinto, Molly Williams, Sheng Xu, Jochen Kruecker, Pingkun Yan, Baris Turkbey, Peter L. Choyke, Bradford J. Wood

**Affiliations:** ^1^Center for Interventional Oncology, National Institutes of Health Clinical Center, Bethesda, MD 20892, USA; ^2^National Cancer Institute, National Institutes of Health, Urologic Oncology Branch, Bethesda, MD 20892, USA; ^3^Walter Reed National Military Medical Center, Bethesda, MD 20814, USA; ^4^Philips Research North America, Briarcliff Manor, NY 10510, USA; ^5^Molecular Imaging Program, National Cancer Institute, National Institutes of Health, Bethesda, MD 20892, USA

## Abstract

In magnetic resonance iimaging- (MRI-) ultrasound (US) guided biopsy, suspicious lesions are identified on MRI, registered on US, and targeted during biopsy. The registration can be performed either by a human operator (visual registration) or by fusion software. Previous studies showed that software registration is fairly accurate in locating suspicious lesions and helps to improve the cancer detection rate. Here, the performance of visual registration was examined for ability to locate suspicious lesions defined on MRI. This study consists of 45 patients. Two operators with differing levels of experience (<1 and 18 years) performed visual registration. The overall spatial difference by the two operators in 72 measurements was 10.6 ± 6.0 mm. Each operator showed a spatial difference of 9.4 ± 5.1 mm (experienced; 39 lesions) and 12.1 ± 6.6 mm (inexperienced; 33 lesions), respectively. In a head-to-head comparison of the same 16 lesions from 12 patients, the spatial differences were 9.7 mm ± 4.9 mm (experienced) and 13.4 mm ± 7.4 mm (inexperienced). There were significant differences between the two operators (unpaired, *P* value = 0.042; paired, *P* value = 0.044). The substantial differences by the two operators suggest that visual registration could improperly and inaccurately target many tumors, thereby potentially leading to missed diagnosis or false characterization on pathology.

## 1. Introduction

Prostate cancer is the second most common cause of cancer-related mortality in men, with a lifetime risk of one in six [1]. The standard of care for prostate biopsy is to obtain 10–14 cores systematically from distributed regions of the prostate using ultrasound (US) imaging and achieves an overall cancer detection rate of ~30–44% [2–6]. Unlike other solid organs, prostate biopsy is not routinely targeted to specific lesions. It can also detect inconsequential microscopic foci of low grade cancer leading to overtreatment in some cases. Magnetic resonance imaging (MRI) offers the ability to localize suspicious lesions within the prostate. In-gantry MRI-guided biopsy is a lengthy and cumbersome procedure and is unlikely to become widespread due to its cost and limited availability of MRI [7]. MRI-US fusion systems address these issues by fusing high resolution MRI to US imaging [8, 9]. By registering previously acquired MRI to US imaging, suspicious areas on MRI are superimposed on the real-time US image to enable targeted biopsy, with real-time adjustment of the biopsy trajectory in relation to codisplayed targets. Multiple commercial solutions exist, and all entail software-based registration of the MRI to the US image. The software registration was shown to be fairly accurate in phantom studies (mean error 2.4 ± 1.2 mm with a maximum error of 4.8 mm) and retrospective clinical evaluations [8]. Moreover, such MRI-US fusion biopsy almost doubled the cancer detection rate of standard 12-core transrectal US (TRUS) biopsy [10]. It was particularly helpful for lesions in the anterior prostate, an area typically undersampled by US guided systematic biopsy [11].

In the absence of software registration, another, simpler and less costly approach is for the operator to mentally approximate the location of the lesion on the US image after studying MR images, a process termed “mental, cognitive, or visual registration.” The diagnostic yield for anterior lesions was greatly improved using this method over freehand targeting, with both a radiologist and a urologist evaluating the MRI [12]. Such visual registration is widespread in image guided biopsy; however it is operator-dependent leading to issues regarding reproducibility. Previous reports showed that software registration and visual registration are comparable with respect to cancer detection rate [13, 14].

The purpose of our study was to assess the capability of visual registration in localizing suspicious lesions between operators with differing levels of experience (<1 and 18 years).

## 2. Materials and Methods

### 2.1. Patient Population

This study was conducted as part of an ongoing institutional review board- (IRB-) approved clinical trial of MRI-US fusion prostate biopsy. Eligible patients had a history of prostate cancer, elevated PSA, or clinical suspicion of prostate cancer and had at least one suspicious lesion visualized on multiparametric MRI. The commercial fusion platform used for this study was the UroNav system (in Vivo Corp, Philips Healthcare, Gainesville, FL). Patients had standard of care 12-core transrectal US extended sextant biopsies and two targeted MRI-US fusion guided biopsies per MRI-identified lesion. Visual registration was performed by two operators from February to June 2013, without actual visual registration-directed biopsies. The study population consists of 45 patients with 2.6 MRI-defined lesions on average (range 1 to 6) (Table 1). Three patients had a previous record of treatment (hormone therapy or focal laser ablation). 16 patients had known lesion(s) of cancer, but the location of cancer was blinded to the operator. One operator performed visual registration in 34 patients and the other in 23 patients, and 12 patients were common to both operators. We note that the choice of the operator who performs visual registration on which patient and target lesion was random.

### 2.2. Multiparametric MRI

**Figure 1:**
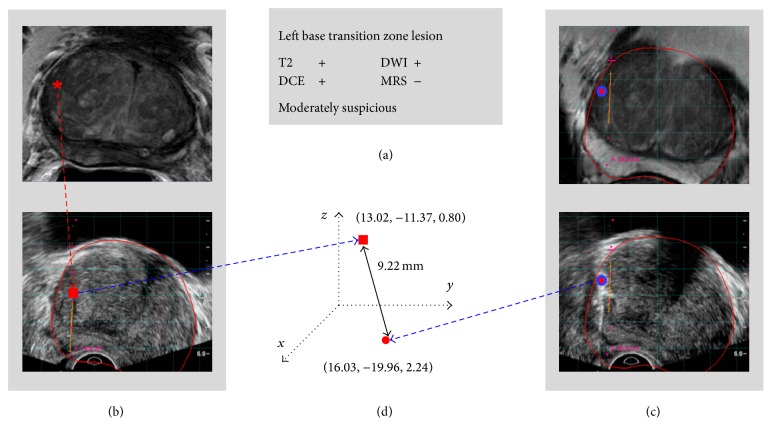
Method for calculating target location difference between visual registration and semiautomated MRI-US fusion. (a) MRI detects lesions (MRI reading). (b) A suspicious lesion is visually correlated and estimated on the US image (red rectangle). (c) The lesion is also registered through MRI-US fusion system (red circle). (d) The spatial difference between the estimated target using just ultrasound and actual target using EM-based MRI-US fusion is calculated. T2: T2-weighted, DWI: diffusion-weighted, DCE: dynamic contrast-enhanced, and MRS: MR spectroscopy.

Using 3-Tesla MRI scanner (Achieva, Philips Medical Systems, Cleveland, OH) with a 6- or 16-channel body coil (SENSE, Philips Healthcare, Cleveland, OH) combined with an endorectal coil (BPX-30, Medrad, Pittsburgh, PA), multiparametric MRI images (T2-weighted, diffusion-weighted, dynamic contrast-enhanced, and MR spectroscopy) were acquired axial to the prostate, orthogonal to the posterior margin of the prostate with respect to rectum. MRIs were independently evaluated (Figure 1) by two experienced genitourinary radiologists (BT, PLC, with 6 and 13 years of experience). The location of the identified suspicious lesions was recorded in an MRI coordinate system and imported into the UroNav fusion system. The criteria for a positive lesion on multiparametric MRI have been previously described [15–17]. The targets defined by multiparametric analysis were marked on the T2-weighted images and displayed on triplanar (axial, sagittal, and coronal) images as point-targets.

### 2.3. MRI-US Fusion

After lesions were identified on MRI, patients underwent MRI-US fusion prostate biopsy using the UroNav system. During the biopsy, an EM field generator was placed above the pelvis and a transrectal 2-dimensional end-fire US probe (Philips C9-5ec) with detachable electromagnetic (EM) tracking sensors was positioned in the rectum. This enables real-time tracking of the US transducer and biopsy guide during the procedure. The operator scanned the prostate from its base to its apex with the tracked probe, and a fan-shaped 3-dimensional volumetric US image was reconstructed which was spatially registered with the annotated prebiopsy T2-weighted images. After registration of the MRI and US coordinate systems, the live US image (iU22, Philips Medical Systems, Andover, MA) was fused with the MRI images in real-time (Figure 1). The MRI and US images were examined from the base to the apex of the prostate on axial and sagittal views for their correspondence. Image registration was maintained despite changes in the position of the TRUS probe by tracking it with an electromagnetic tracking system (In Vivo, Gainesville, FL, Philips Interventions, formerly Traxtal Inc., Toronto, Ontario, Canada, and Northern Digital Inc., Waterloo, Canada) as previously described [8].

### 2.4. Visual Registration

Two operators performed visual registration: an interventional radiologist with over 18 years of experience (Bradford J. Wood) and a urology resident with 6 months of experience (Molly Williams). After registration of MR and US images, each operator independently and sequentially attempted to locate the MRI-defined lesion on the live US image while blinded to the MRI-US fusion image (Figure 1). The operator was allowed to review the target location on triplanar MRI images. Each operator was blinded to the other operator's visual registration. After the target was determined using just the US image, the image was frozen and the target's location, trajectory, and depth were recorded in the MRI coordinate system based on the prior semiautomated software registration. The locations of the suspicious lesion on the prebiopsy MRI and on the real-time US by visual registration were available in the MRI coordinate system. The Euclidean distance was employed to compute the spatial distance between the estimated target location based upon visual registration and actual target location based upon software-based MRI-US fusion registration (Figure 1). Since the MRI-defined lesions and visually identified lesions are modeled as point targets, the distance was computed between two points in the MRI coordinate system. No actual biopsy was performed on the visually identified targets. Following visual registration, one of the operators sequentially performed two targeted MRI-US fusion guided biopsies per MRI-identified lesion and 12-core US sextant biopsies.

### 2.5. Statistical Analysis

Data analysis was performed using R software version 2.15.2 (GNU General Public License). Statistical significance of the spatial difference in visual registration between two operators was determined by the two-tailed paired and unpaired* t*-tests. Spearman's rank correlation was used to assess the correlation between the spatial distances in visual registration. A significance level of 0.05 was used for all statistical testing.

## 3. Results

### 3.1. Visual Registration Differs from Software Registration

The two operators performed visual registration in 45 patients: 39 MRI-defined lesions from 34 patients by operator 1 (OP1; 18-year experience; Bradford J. Wood) and 33 MRI-defined lesions from 23 patients by operator 2 (OP2; 6-month experience; Molly Williams). Each operator performed the visual registration once per lesion and the Euclidean distance between the designated MR target and visually identified target by the two operators was computed (Table 2). For these 72 measurements, the mean spatial distance was shown to be 10.6 ± 6.0 mm with a maximum distance of 26.0 mm. Only 15.3% of the cases were <5 mm away from the MR targets, and the spatial distance of >20 mm was obtained for 12.5%. Moreover, the mean distance was substantial regardless of the location of suspicious lesions, 14.9 ± 7.1 mm in the base and 9.4 ± 5.2 mm in the apex, but significantly higher in the base (*P* value = 0.004). In the peripheral zone, the distance was significantly lower than that of the central gland (peripheral zone = 9.2 ± 4.6 mm, central gland = 13.4 ± 7.3 mm, *P* value = 0.004). Regarding the pathology result of the targeted MRI biopsy on the same lesion, there was no significant spatial difference between the benign (10.5 ± 5.5 mm) and tumor (11.1 ± 7.3 mm) lesions (*P* value = 0.7). The distance did not appear to be systematic (i.e., predictable).

### 3.2. Performance of Visual Registration Is Operator-Dependent

**Figure 2:**
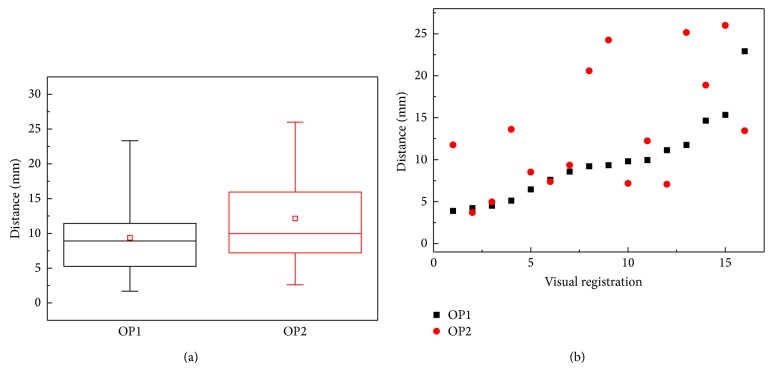
Spatial distances between visually identified targets and software registration defined targets. (a) Relative variability and variation of visual registration by two operators in 16 lesions. (b) Spatial distances by the two operators were plotted. The distances were sorted by the distance of OP1. More experienced operator (OP1) shows better reproducibility.

Visual registration between the two operators was compared. For the 39 and 33 measurements, the spatial distances of 9.4 ± 5.1 mm and 12.1 ± 6.6 mm were obtained by OP1 and OP2, respectively. For only 12 patients with 16 lesions, both operators were able to perform visual registration (32 measurements combined). In a head-to-head comparison of these 16 lesions (Figure 2(a)), OP1 and OP2 showed the spatial distances of 9.7 ± 4.9 mm and 13.4 ± 7.4 mm, respectively. For both comparisons, the differences were significant (unpaired, *P* value = 0.042; paired, *P* value = 0.044). Moreover, the performance of the operators was dependent on the location of suspicious lesions (Table 2). For the 39 and 33 measurements, both operators performed a better visual registration for the lesions in the apex (OP1: 8.8 ± 5.3 mm, OP2: 10.1 ± 5.2 mm) than in the base (OP1: 11.8 ± 5.9 mm, OP2: 19.0 ± 6.9 mm). They also showed a lower spatial distance in the peripheral zone (OP1: 8.3 ± 3.8 mm, OP2: 10.2 ± 5.2 mm) than in the central gland (OP1: 11.3 ± 6.6 mm, OP2: 16.1 ± 7.5 mm). The same trends have been observed for the 12 patients with 16 lesions where both operators performed visual registration (Table 2). Also, no significant difference was found between benign and tumor lesions for the two operators.

Though better registration was shown by the more experienced operator, the distances by both operators were substantial and may lead to inaccurate biopsies (4- to 5-fold larger than the software registration error of 2.4 ± 1.2 mm). Further, the spatial distances obtained by the two operators were correlated to each other (*ρ* = 0.532, *P* value = 0.036) as shown in Figure 2(b). As the distance of OP1 increases, OP2 also showed higher distance that was generally larger than that of OP1. In addition, the visually defined targets by the two operators were substantially distant from each other (11.0 ± 5.3 mm).

## 4. Discussion

The results of this study show that although visual registration could provide a reasonable approximation in some cases, a substantial distance to an actual target lesion was seen in the majority of cases which could not be overcome by experience. Spatial disparity related to visual registration was substantial in regard to the typical tissue core length of 10–20 mm. This implies that if visual registration is employed routinely, many tumors could be improperly or inaccurately targeted, potentially leading to missed diagnoses or false characterizations on pathology.

Furthermore, the high variance of spatial distance to a designated MR target suggests that many targets may be completely missed even with experienced operators (since the distance goes up with both experienced and inexperienced operators in the same more difficult cases). This would adversely affect the rate of cancer detection and would disproportionately affect smaller targets. For some cases, the spatial distance may not have a direct bearing on cancer detection since biopsy results are also dependent on the size of the lesion, the needle trajectory, and the plane of the spatial disparity of visual registration. However, the ability to more accurately target the center or most suspicious region of the lesion (as would occur with more accurate fusion techniques) is certainly lost with visual registration resulting in less accurate grading and potentially more upgrading at surgery. Note that this study models the MR-visible lesions as point targets, and so lesion size does not affect this assessment of distance, and the impact of lesion size is thus not reflected here.

**Figure 3:**
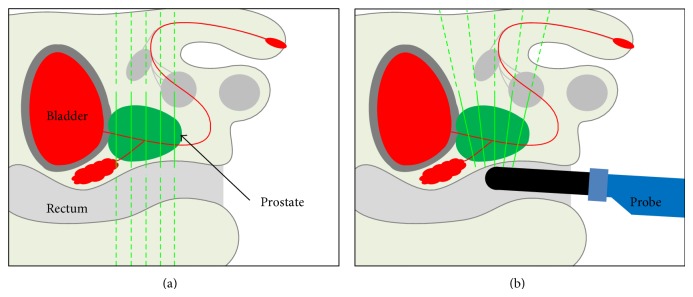
Different planes (yellow lines) complicate visual registration. (a) MRI and (b) TRUS slice the 3D prostate volume into different planes of 2D images, based upon different and variable orientations.

Visual registration might be improved when various anatomical landmarks or features such as calcifications and cysts and hypoechoic area are visible to provide reference points. Such anatomic landmarks, in the absence of gland deformation, could improve the reliability of visual registration. However, the different planes with which MRI and US are obtained are a complicating factor and are difficult for the human brain to account for. For example, the TRUS 2D images planes arise in a fan-shaped pattern instead of arising from parallel planes (Figure 3). These fanned planes on end-fire TRUS are markedly different than the axial anatomic imaging plane on MRI, which makes visual registration difficult and dependent upon organ shape. The imaging plane disparity becomes most evident in the anterior base and anterior apical lesions, which may appear in unexpected locations on anatomic axial MRI, versus fan-beam shaped TRUS. Moreover, deformation of the prostate caused by the endorectal coil and US transducer may be different in character, further complicating visual registration. The effect of such deformation may be minimal using nonendorectal coil MRI.

Freehand targeted biopsies direct biopsies roughly towards the anatomic section of the prostate where the lesion was visualized on MRI. Although detection rates likely improve over conventional blind biopsy, the effectiveness of the freehand visual registration technique is still restricted to the preciseness of the anatomic section and the capability of the operator to pinpoint the section and the target, as well as to convert the fanned oblique planes of 2D TRUS into the axial plane of MRI. In other words, challenging areas to visualize or recognize are still likely to have lower diagnostic yield. However, these are the very lesions that should be detected because they are often missed by conventional systematic biopsy. The higher spatial distance to a targeted lesion in the base (14.9 ± 7.1 mm) than in the apex (9.4 ± 5.2 mm) as well as in the central gland (13.4 ± 7.3 mm) than in the peripheral zone (9.2 ± 4.6 mm) of the prostate suggests that the performance of visual registration is dependent upon the location of suspicious lesions.

The spatial distance was larger for the less experienced operator. Improvement seen with the experienced operator may indicate there is a learning curve for visual registration. Visual registration is error-prone even for experienced operators, suggesting an inherent limitation of the technique. Further, the visually identified lesions by the inexperienced and experienced operators were likely located at a considerably longer distance away from the actual targets. This implies that the biopsy samples could be taken from completely different locations, depending on the operator, even though they were targeting the same lesion.

These shortcomings of visual registration could be minimized by software registration which is inherently a more standardized process, less prone to human input and human error. Due to the whole volumetric registration of the prostate, it could aid in sampling the exact area of the targeted lesion at biopsy [18, 19]. Even, the technique does not require anatomical landmarks to provide reference points. The error reported with software registration is 2.4 ± 1.2 mm [8], which is 4- to 5-fold smaller than the spatial disparity by visual registration. Since the error was measured in phantom studies, it may differ from that with real patients' MRI and US images. However, the retrospective clinical evaluation ensures its accuracy and robustness [8]. In practice, software registration is examined and confirmed for the correspondence between MRI and US image by the operators. A substantial error in registration will be recognized and corrected prior to visual registration and targeted and sextant biopsies.

There are several limitations in this study. First, through the software-based fusion platform, a visually registered target is converted into an MR coordinate and used to estimate spatial distance. That is, software registration is the tool used to obtain the trajectory data during visual registration. Both the visually defined target and software registration directed target use the software-based fusion platform for measuring the data, without recording errors of the fusion system itself. Errors generated by software registration may, therefore, affect visual registration and spatial distance estimation. However, compared to the larger spatial distance of visual registration, its contribution may be insignificant. Second, prostate deformation by the US probe and patient motion can affect the performance of visual registration and was not accounted for in this study. Third, only two operators performed visual registration. A large-scale study should be conducted to confirm the differences among operators with differing levels of experience. Also, operators' specialty and familiarity with the biopsy system may affect visual registration. Fourth, the number of patients and lesions are limited in this study. Depending on the patient cohort, the characteristics of the lesions in MRI and US images may vary. An extended study should be followed to confirm the findings of this study. Last, the location of the MRI-US fusion target is used as the gold-standard comparison. The quality of visual registration is only evaluated as it differs from the corresponding MR target. Future studies could further characterize cancer detection rate and Gleason grades found on visually registered biopsies regarding the distance to a target and the experience level of operators.

## 5. Conclusion

Here, we studied the ability of visual registration to locate suspicious lesions defined on MRI with respect to semiautomated software registration in MRI-US guided biopsy. The substantial spatial differences by the both experienced and inexperienced operators were observed, suggesting an inherent limitation of visual registration that cannot be overcome by experience. The spatial disparity, in practice, could cause improper and inaccurate localization of targets differing from the software registration defined targets.

## Figures and Tables

**Table 1:** Characteristics of patient cohort.

	OP1 ∪ OP2	OP1	OP2	OP1∩OP2
Patient, *n*	45	34	23	12
Age at biopsy, mean ± SD	62.3 ± 8.1	63.1 ± 7.7	62.3 ± 8.3	64.6 ± 7.2
PSA ng/mL, mean ± SD	7.8 ± 8.4	7.7 ± 9.2	8.3 ± 5.3	8.4 ± 4.9

Lesion, *n*	56	39	33	16
PZ, *n* (%)	38 (68)	25 (64)	22 (67)	9 (56)
CG, *n* (%)	18 (32)	14 (36)	11 (33)	7 (44)
Apex, *n* (%)	31 (55)	19 (49)	19 (58)	7 (44)
Mid, *n* (%)	15 (27)	12 (31)	8 (24)	5 (31)
Base, *n* (%)	10 (18)	8 (20)	6 (18)	4 (25)
Benign, *n* (%)	41 (73)	30 (77)	24 (73)	13 (81)
Tumor, *n* (%)	15 (27)	9 (23)	9 (27)	3 (19)

OP1 and OP2 indicate the more experienced and less experienced operators, respectively. *n* and SD denote a number and standard deviation, respectively. OP1 ∪ OP2 denotes that both or either of OP1 and OP2 performed visual registration. OP1∩OP2 indicates that both OP1 and OP2 performed visual registration.

**Table 2:** Performance of visual registration.

	OP1 ∪ OP2	OP1	OP2	OP1∩OP2	
				OP1	OP2
All Lesions, mean ± SD (mm)	10.6 ± 6.0	9.4 ± 5.1	12.1 ± 6.6	9.7 ± 4.9	13.4 ± 7.4
PZ, mean ± SD (mm)	9.2 ± 4.6	8.3 ± 3.8	10.2 ± 5.2	7.4 ± 2.8	10.0 ± 6.3
CG, mean ± SD (mm)	13.4 ± 7.3	11.3 ± 6.6	16.1 ± 7.5	12.5 ± 5.8	17.7 ± 6.7
Apex, mean ± SD (mm)	9.4 ± 5.2	8.8 ± 5.3	10.1 ± 5.2	7.0 ± 2.8	9.4 ± 3.2
Mid, mean ± SD (mm)	10.0 ± 5.4	8.6 ± 4.0	11.9 ± 6.6	9.3 ± 3.7	12.7 ± 8.6
Base, mean ± SD (mm)	14.9 ± 7.1	11.8 ± 5.9	19.0 ± 6.9	14.8 ± 6.0	21.3 ± 5.7
Benign, mean ± SD (mm)	10.5 ± 5.5	9.4 ± 4.6	11.9 ± 6.3	10.0 ± 5.3	12.9 ± 7.3
Tumor, mean ± SD (mm)	11.1 ± 7.3	9.4 ± 6.8	12.7 ± 7.6	8.2 ± 3.4	15.4 ± 9.0

OP1 and OP2 indicate the more experienced and less experienced operators, respectively. Mean and SD denote average and standard deviation in mm, respectively. OP1 ∪ OP2 denotes that both or either of OP1 and OP2 performed visual registration. OP1∩OP2 indicates that both OP1 and OP2 performed visual registration.
